# Genomic divergence and cohesion in a species of pelagic freshwater bacteria

**DOI:** 10.1186/s12864-017-4199-z

**Published:** 2017-10-16

**Authors:** Matthias Hoetzinger, Martin W. Hahn

**Affiliations:** Research Institute for Limnology, University of Innsbruck, Mondseestrasse 9, A-5310 Mondsee, Austria

**Keywords:** *Polynucleobacter*, Freshwater bacteria, Homologous recombination, Gene flow, Population structure, Genomic cohesion

## Abstract

**Background:**

In many prokaryotic genera a clustered phylogeny is observed, akin to the occurrence of species in sexually reproducing organisms. For some taxa, homologous recombination has been invoked as the underlying mechanism providing genomic cohesion among conspecific individuals. Whether this mechanism is applicable to prokaryotes in freshwaters with low habitat connectivity – i.e. elevated geographic barriers to gene flow – is unclear. To investigate further we studied genomic trends within the globally abundant PnecC cluster (genus *Polynucleobacter*, *Betaproteobacteria*) and analyzed homologous recombination within the affiliated species *P. asymbioticus*.

**Results:**

Comparisons among 20 PnecC genomes revealed a clearly discontinuous distribution of nucleotide sequence similarities. Among the nine conspecific individuals (*P. asymbioticus*) all average nucleotide identity (ANI) values were greater than 97%, whereas all other comparisons exhibited ANI values lower than 85%. The reconstruction of recombination and mutation events for the *P. asymbioticus* core genomes yielded an r/m ratio of 7.4, which is clearly above estimated thresholds for recombination to act as a cohesive force. Hotspots of recombination were found to be located in the flanking regions of genomic islands. Even between geographically separated habitats a high flux of recombination was evident. While a biogeographic population structure was suggested from MLST data targeting rather conserved loci, such a structure was barely visible when whole genome data was considered. However, both MLST and whole genome data showed evidence of differentiation between two lineages of *P. asymbioticus*. The ratios of non-synonymous to synonymous substitution rates as well as growth rates in transplantation experiments suggested that this divergence was not selectively neutral.

**Conclusions:**

The high extent of homologous recombination among *P. asymbioticus* bacteria can act as a cohesive force that effectively counteracts genetic divergence. At least on a regional scale, homologous recombination can act across geographically separated ecosystems and therefore plays an important role in the evolution and consistency of bacterial freshwater species. A species model akin to the biological species concept may be applicable for *P. asymbioticus*. Nonetheless, two genetically distinct lineages have emerged and further research may clarify if their divergence has been initiated by reinforced geographical barriers or has been evolving in sympatry.

**Electronic supplementary material:**

The online version of this article (10.1186/s12864-017-4199-z) contains supplementary material, which is available to authorized users.

## Background

Diversity within many prokaryotic genera is not continuous but clustered. This is evidenced by sequencing of genomes [1–3], metagenomic DNA [4–7] and single genes [8–10]. Considering average nucleotide identity (ANI), there is a noticeable sparsity of values between 82 and 96% [11]. For many taxa this “ANI-gap” has been proven useful for species delineation on a genomic basis; that is, the upper bound of the gap coincides with suggested thresholds of 95–96% ANI [11–13]. It can be assumed that there exists an underlying biological process that is maintaining genomic coherence. Two important mechanisms have been proposed that can eventually promote genomic similarity among individuals: periodic selection [14, 15] and homologous recombination (HR) [16, 17]. In the periodic selection model the evolving diversity within populations is periodically purged by selective sweeps [18, 19]. In selective sweeps, possibly certain loci only but not entire genomes hitchhike to high frequencies along with selectively beneficial mutations, which may be the consequence of recombination unlinking different regions of the genome [7, 20, 21]. However, even without the immediate action of selection, HR can reduce intra-lineage divergence [17, 22, 23]. This mechanism of cohesion is consulted in the biological species concept [24]. It is still a matter of debate if or rather to what extent this concept is applicable to microbes [25–28]. Generally, recombination can provide cohesion for a species only if it spreads genetic variability faster than it is accumulated by mutation. Theoretical models based on neutral Fisher-Wright populations, which consider an exponential decrease of HR rate with sequence divergence according to experimental data [29–31], suggest a threshold for recombination rate relative to mutation (r/m), above which population divergence is prevented [17, 22, 23]. This threshold is passed at r/m ratios between 0.25 and 2 according to [17], i.e. populations with lower ratios are free to diverge clonally, whereas above the threshold divergence is constrained by the cohesive effects of HR.

Prokaryotic HR has been widely studied in pathogenic bacteria, where it is often linked to antibiotic resistance [32, 33]. Data from multilocus sequence typing (MLST) have proved helpful in assessing the degree of HR in various populations of bacteria [34, 35]. The estimated extent of recombination varies drastically between different prokaryotic taxa [36–38]. The estimated r/m ratio of 63.1 for marine SAR11 isolates is one of the highest recorded for bacteria [36]. Various studies invoke HR within lineages as a cohesive force, which may result in speciation of ecologically differentiated lineages [20, 39–41]. In contrast, there are examples of monomorphic pathogens for which highly clonal population structures are suggested [42–45]. Exceptionally low recombination frequencies have also been reported for the freshwater clade of SAR11, estimated to be more than two orders of magnitudes lower than in the marine sister group [36, 46, 47]. The low recombination rates detected for freshwater SAR11 are supposedly the result of purged diversity following the transition from marine to freshwater systems [46]. Beyond that, the geographic separation of inland waters may have an important effect on HR rates and speciation. While many freshwater habitats are connected by stretches of running water, others can be largely isolated from other water bodies [48]. In the latter case, prokaryotic gene flow across distant habitats is dependent on dispersal by air or animals. The availability of stepping stones can increase the connectivity across distant habitats [49]. If stepping stones are missing, populations may evolve in isolation, as has been shown for thermoacidophilic archaea [50, 51]. However, biogeographical isolation has been observed even in microbial species with less restrictive habitat requirements and may enforce allopatric speciation [52, 53]. The role of HR in giving coherence to prokaryotic freshwater species is widely unclear. Here we studied this issue in *Polynucleobacter* bacteria affiliated with the PnecC cluster.

PnecC bacteria are abundant in the pelagic zone of diverse freshwater habitats [54–57] and show cosmopolitan distribution [58]. PnecC represents a cryptic species complex, including a few described and an unknown number of undescribed species [59–61]. *Polynucleobacter asymbioticus*, formerly termed the F10 lineage [48, 56], has so far been detected only in central Europe. The species is particularly abundant in dystrophic ponds in the Austrian Alps, where it accounted for up to 46% of total bacterioplankton and was shown to maintain persistent populations [48, 62]. Isolates, which cannot be obtained by standard cultivation methods [63], are available from only three sites (Loibersbacher Höhe (Loi), Rauriser Urwald (Rau), Trög (Tro)) within a maximum distance of 76 km separating the three sites [64]. At each site there are several dystrophic ponds not directly connected to other water bodies by running water. While numerous ecologically similar ponds are present throughout the Alps, such ponds are rare in the surrounding lowlands. The Loi site, at the foothills of the Alps, therefore represents the periphery of the *P. asymbioticus* range. The habitat range of *P. asymbioticus* and other PnecC species/lineages has been shown to be restricted, as strains isolated from acidic water were unable to grow in alkaline water and vice versa [59]. This may strengthen geographic barriers for PnecC bacteria, which calls into question whether species coherence can be maintained among subpopulations from distinct sites. For the flexible gene pool of *P. asymbioticus* it has been shown that gene flow across geographically separated sites is realized. This gene flow is mainly attributed to illegitimate recombination of genomic islands, which can even be exchanged across species boundaries and may often be mediated by phages [64]. The exchange of genomic islands is a major driver for diversification regarding the flexible genome of the species. In contrast, the extent of HR in the core genome potentially mediating species coherence is unclear and was investigated in this study.

## Results

### Genome clustering in PnecC

**Fig. 1:**
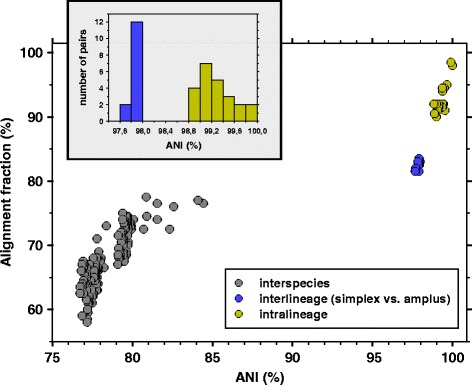
Average nucleotide identities in PnecC. Alignment fraction plotted against ANI for pairwise comparisons of 20 PnecC genomes. The insert at the top left shows the number of genome pairs at 0.2% ANI intervals for the intraspecific comparisons

Twenty strains affiliated with the PnecC cluster, nine of which representing *P. asymbioticus*, have been whole genome sequenced in previous studies [60, 64–70] (Additional file 1). These genomes were analyzed for the distribution of pairwise ANI values (Fig. 1, Additional file 2). A phylogenetic tree based on partial 16S rRNA gene sequences of the used and related strains is shown in Additional file 3. No ANI values in the range of 85% - 97% are found. The values above 97% ANI represent intraspecies comparisons corresponding to the nine *P. asymbioticus* genomes and two genomes affiliated with a *Polynucleobacter* species which has not yet been described. A separation into two lineages, simplex and amplus [64], is visible for *P. asymbioticus*. Each other genome analyzed here represents a putatively distinct species, several of which have been described [59–61]. It is evident that different species within the cryptic species complex PnecC are genomically well differentiated. Potential mechanisms responsible for the coherence within the species *P. asymbioticus* are discussed in the following.

### HR and population differentiation inferred from MLST and genomic data

**Fig. 2:**
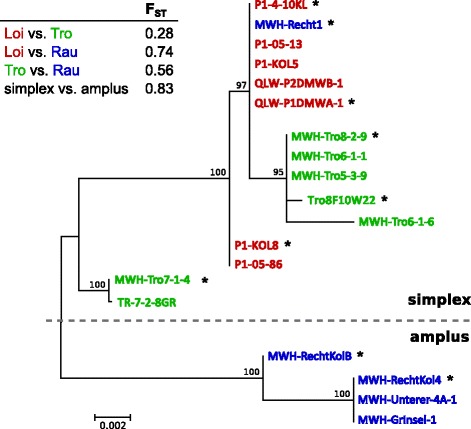
Unrooted maximum likelihood tree from MLST data of *P. asymbioticus* isolates. From each sampling only one representative of each sequence type was included, to avoid the possible effects of transient blooming of certain genotypes. The F_ST_ data refers to the same set of strains shown in the tree. Genome sequenced strains are indicated by stars

The 37 *P. asymbioticus* strains used in this study have been isolated over eleven years [64]. Removal of identical MLST sequence types obtained from single samplings resulted in a remaining data set of 19 strains. Subpopulations clustered according to the three different sites of origin are all significantly differentiated in MLST data (Fig. 2). The fixation index (F_ST_) is highest between Loi and Rau (0.74), medium between Tro and Rau (0.56), and lowest between Loi and Tro (0.28). The r/m ratio estimated by ClonalFrame [71] is 1.34 (Table 1), i.e. the estimated number of polymorphisms arisen from HR is higher than the one from mutation. If only strains affiliated with the simplex lineage are included in the analysis, the r/m ratio increases to 4.08.

**Table 1:** Results of the ClonalFrame analysis

Data	STs / genomes	# Sites	δ	r / m	π	μ / ρ
MLST	7 simplex, 2 amplus	7259	662 [249–1636]	1.3 [0.5–2.7]	0.0126	0.0094 [0.0047–0.0245]
MLST	7 simplex	7259	609 [230–1498]	4.1 [1.5–9.1]	0.0073	0.0018 [0.0008–0.0048]
genomes	7 simplex, 2 amplus	1,814,913	319^a^ [305–333]	7.4 [6.9–7.8]	0.0111	0.0015 [0.0016–0.0014]

*STs* sequence types, *δ* average tract length of recombination events, *r/m* ratio of probabilities that a given site has been altered through recombination and mutation, *π* average nucleotide diversity per site, *μ/ρ*, ratio of mutation to recombination calculated as π (r/m)^−1^ according to Fig. 4 in [23]. 95% confidence intervals are given in brackets

^a^As a result of the ClonalOrigin analysis a median δ of 2340 was obtained

**Fig. 3:**
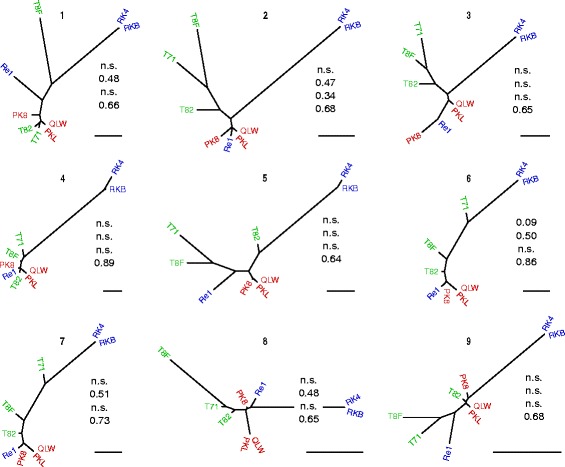
Unrooted RAxML trees for different segments of the core genome alignment. The scale bars indicate nucleotide divergence of 0.005. F_ST_ values in the same order as given in Fig. 2 are shown for each tree. n.s., not significant (*p* > 0.1); QLW, QLW-P1DMWA-1; PKL, P1–4-10KL; PK8, P1-Kol8; Re1, MWH-Recht1; RKB, MWH-RechtKolB; RK4, MWH-RechtKol4; T71, MWH-Tro7–1-4; T82, MWH-Tro8–2-9; T8F, Tro8F10W22

The r/m values estimated for nine strains from whole genome data are 7.4, i.e. higher than those estimated from MLST data (Table 1). In order to see whether the high HR rates inferred would be reflected in incongruent phylogenies of different genome segments, the core genome alignment was exemplarily split into nine sections of equal length. The nine respective phylogenetic trees were all incongruent to each other (Fig. 3), which was assessed with the Shimodaira-Hasegawa test [72]. The test rejects the null hypothesis for all combinations of trees and alignment data with *p*-values lower than 0.001, which certainly confirms incongruence among all trees [73]. Differences in log-likelihood scores for different tree topologies with respect to certain sequence data are given in Table 2. Figure 3 shows that the remote position of the two amplus genomes is rather conserved in all nine trees. This suggests that HR between the lineages may be less frequent than within the simplex lineage. This is also evident from the higher r/m ratio inferred from the MLST data when strains of the amplus lineage are excluded from the analysis. In any case, the r/m and μ/ρ ratios, respectively, estimated from MLST as well as genomic data imply that HR can act as a cohesive force within *P. asymbioticus* according to theoretical models [17, 23]. The strains used in the analysis were isolated from distant sites. Consequently it is suggested that the cohesive effect of HR can act across distant sites, counteracting divergence of the species at least on this geographic scale (76 km max. Distance). Nonetheless, population differentiation between sites is evident to some extent in genomic data (Fig. 3) and to a greater extent in the MLST data (Fig. 2). The simplex and amplus lineages are well differentiated with respect to all sequencing data.

**Table 2:** Shimodaira-Hasegawa test for incongruence among tree topologies for different segments of the core genome alignment

Segment	1	2	3	4	5	6	7	8	9
1	–	10,378	17,700	26,494	11,041	12,955	15,541	8745	12,441
2	7100	–	1908	11,511	4635	4823	5961	5295	8089
3	13,765	5472	–	13,087	10,540	15,234	10,690	10,714	10,508
4	14,493	11,159	7432	–	6411	9415	8898	12,331	13,047
5	13,057	8894	12,004	16,317	–	14,431	10,825	12,648	11,782
6	8979	7055	8418	5340	8223	–	2023	9517	8523
7	15,661	10,081	10,433	6309	11,983	4014	–	13,848	15,287
8	3819	3428	4378	8328	5980	3416	3095	–	3069
9	6474	4660	6312	9181	3951	4865	4985	2889	–

For the sequence data of each segment (rows), the differences in log-likelihood scores between the original tree topology and each other tree topology (columns) are given.

*P*-values for the differences are all lower than 0.001

### Lack of mismatch repair system

Mismatch repair systems have been shown to prevent HR in various taxa [74–77]. All 20 PnecC genomes were analyzed for mismatch repair systems, and strikingly, *mutS* and *mutL* are missing in all PnecC genomes. In order to check whether this would be a rather unique characteristic of PnecC, or if related taxa would share the absence of *mutS* and *mutL*, the IMG/ER public database was screened for genomes of the genera most closely related to *Polynucleobacter*, i.e. *Cupriavidus* and *Ralstonia* [78]. After removal of single-cell genomes, 29 *Cupriavidus* and 65 *Ralstonia* genomes were obtained. Both *mutS* and *mutL* were found in all 65 *Ralstonia* and in 28 of the 29 *Cupriavidus* genomes. As well, *mutS* and *mutL* were found in *Polynucleobacter* genomes not affiliated with PnecC, i.e. *P. rarus* [79] and *P. cosmopolitanus* [80], but both genes are missing in *P. acidiphobus* [81] (unpublished data).

### Flanking regions of replacement genomic islands as hotspots of HR

**Fig. 4:**
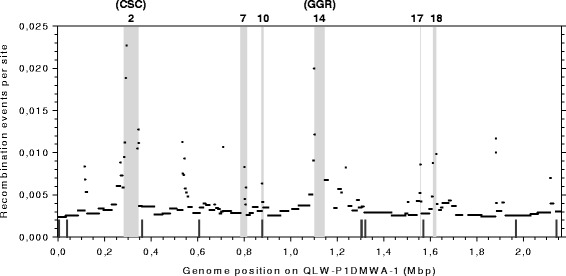
Frequency of recombination with respect to genome position. The y-axis indicates the frequency of recombination events as inferred by ClonalOrigin for each block in the core genome alignment of the nine *P. asymbioticus* strains. The x-axis shows the respective genome position, exemplarily for strain QLW-P1DMWA-1. Genomic islands which have been assigned to the replacement type in [64], i.e. presumably resulted from HR between the flanking sequences of the islands, are highlighted by grey areas and numbered according to their genomic position defined earlier (Fig. 3C in [64]). CSC (cell surface composition) and GGR (giant gene region), designated as in [64], are typical examples of replacement genomic islands (cf. [83]). The position of the loci used for MLST is indicated by short lines at the bottom of the graph

The estimated frequency of HR events, inferred from the core genome alignment of the nine *P. asymbioticus* strains was plotted against the genome position, exemplarily on QLW-P1DMWA-1 (Fig. 4). As the synteny in the core genome of *P. asymbioticus* is well conserved, the figure looks similar if any other of the nine strains is used as reference for genome position. Merely, the x-axis shifts due to differing numbers and sizes of genomic islands among the strains (cf. [64]). Several regions with clearly elevated HR frequencies are apparent. Some of these regions correspond to the flanking regions of six genomic islands which were presumed to be the result of HR earlier [64]. The numbering of these genomic islands in Fig. 4 corresponds to their genomic position assigned in [64]. In particular, the borders of two genomic islands are clearly represented as HR hotspots here. It has been suggested in [64] that the island designated as CSC (cell surface composition) may be involved in the synthesis of exposed structures of the cell surface. Similar genomic islands are found in various other prokaryotes [82]. The other island, designated as GGR (giant gene region), has been shown to contain a giant gene with sequence lengths up to 42 kbp. CSC and GGR are typical examples of replacement genomic islands according to the definition in [83]. In replacement genomic islands relatively big DNA fragments are assumed to be transferred by single recombination events, where homology is required only for the ends of these fragments [82]. It is worth noting that, although the hotspots show clearly elevated per site HR rates, their contribution to the total number of inferred HR events is minor, as they are allocated to relatively short blocks/genomic regions only (Additional file 4).

The loci regarding the MLST data are also indicated in Fig. 4. It is evident that these loci mostly correspond to regions with relatively low recombination frequencies, except for the locus at about 0.88 Mbp genome position, which represents a core gene located within a genomic island.

### HR among different branches of the genealogy

**Fig. 5:**
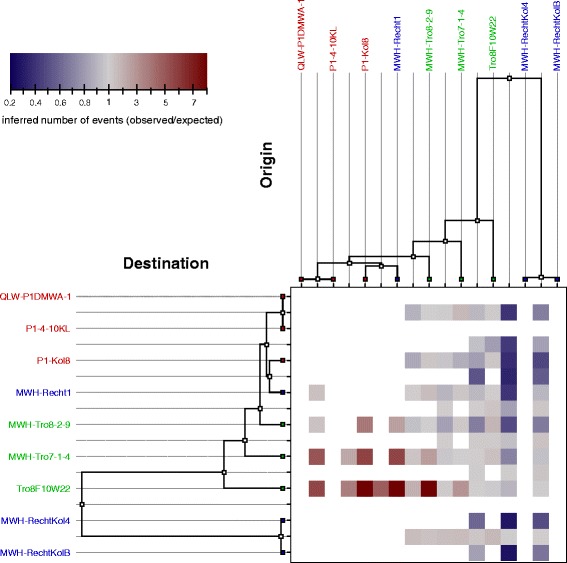
Flux of recombination. Heat map showing the number of recombination events inferred by ClonalOrigin relative to its expectation under the prior model for each donor/recipient pair of branches. Cells for which both the number of observed and expected events are less than three are left white. The underlying numbers are given in Additional file 5

The numbers of observed HR events among all branches of the genealogy relative to the expected numbers under the prior model were inferred by ClonalOrigin [84]. The results are shown in Fig. 5 and the underlying numbers are given an Additional file 5. It is conspicuous that the flux of HR from near ancestors of Loi genomes towards the branches leading to the Tro genomes was clearly enhanced in comparison to the expectations. On the other hand, the flux in the reverse direction was approximately as expected. Another distinctive feature is that recombinational export from the branch leading to the amplus genomes was found less frequently than under the prior model, whereas observed import to that branch was slightly higher than expected. The terminal branches of the two amplus genomes exhibited reduced import from the ancestral branch of the simplex genomes.

### Purifying selection in *P. asymbioticus*

**Fig. 6:**
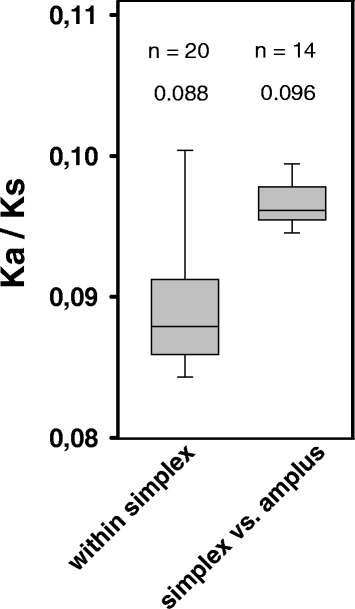
Boxplot of K_a_/K_s_ values. The middle lines indicate median values, the outer lines of the boxes represent the 25th and 75th, and the whiskers the 5th and 95th percentiles. Sample sizes (n) and median values are given above the plots. There is a statistically significant difference between the median values of the two groups shown according to the Mann-Whitney rank test (*p* ≤ 0.001). The K_a_/K_s_ between the two almost identical simplex genomes (QLW-P1DMWA-1 and P1–4-10KL) is 6.6 and was excluded from these analyses. The K_a_/K_s_ between the two amplus genomes is 0.092 (Additional file 6)

K_a_/K_s_ ratios regarding the 1820 core genes of the nine *P. asymbioticus* genomes show that these genomes diverged predominantly under purifying selection (Fig. 6, Additional file 6). Only the two almost clonal strains QLW-P1DMWA-1 and P1–4-10KL exhibit a markedly higher K_a_/K_s_ value of 6.6, which indicates positive selection. It should be noted that relatively high K_a_/K_s_ values have frequently been reported for very closely related strains, which can often be explained by divergence times that have been too short for purifying selection to act on detrimental non-synonymous mutations [85, 86]. However, between the genomes of QLW-P1DMWA-1 and P1–4-10KL there are 19 polymorphisms in coding regions, only two of which are non-synonymous. This ratio is very unlikely to arise in the absence of selection. Disregarding this exception, the median K_a_/K_s_ values for pairwise comparisons within the simplex lineage are significantly lower than the values between the simplex and amplus lineages. Generally, in the presence of purifying selection only, K_a_/K_s_ values tend to decrease with divergence time [85, 86], which would rather favor an opposite trend. It is likely therefore that the divergent evolution of the two lineages has been accompanied by positive selection to some extent. No statistically significant difference in the K_a_/K_s_ ratios is found if they are tested in view of local adaptation, i.e. when ratios within sites are compared to ratios between sites.

### Transplantation experiment

**Fig. 7:**
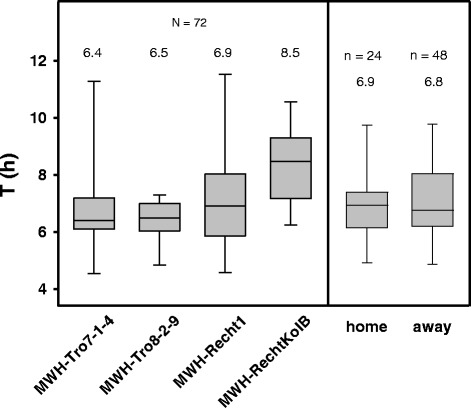
Boxplot of doubling times from the transplantation experiments. The middle lines indicate median values, the outer lines of the boxes represent the 25th and 75th, and the whiskers the 5th and 95th percentiles. Sample sizes (n) and median values are given above the plots. The home group includes doubling times of strains grown in water collected from the same habitat that they were isolated from. The away group includes doubling times of strains grown in water collected from different habitats than those they were isolated from. There is a statistically significant difference between the median doubling times of strain MWH-RechtKolB and all other strains according to the Mann-Whitney rank test (*p* < 0.05). The differences between median doubling times of the other strains as well as between the groups home and away are not significantly different (*p* > 0.05). The underlying growth curves are shown in Additional file 7

The growth potential of four *P. asymbioticus* strains in their home and in two foreign environments was tested by reciprocal transplantation experiments. The pH of the water from Rechteckteich, Trog-7 and Trog-8 was 5.0, 5.6 and 4.7, respectively, in the first experiment (August 2014) and 5.3, 5.6 and 4.9, respectively, in the second experiment (September 2014). All strains were able to grow in all habitats at both times of the year. The bacterial numbers multiplied on average by a factor of 42 and 14 during the 48 h of the first and second experiment, respectively. Growth curves are shown in Additional file 7. Box plots of all determined growth rates for the exponential growth phase are shown in Fig. 7. No statistical differences were found in comparisons of growth rates between habitats or between dates. Comparisons of “home” and “away” likewise yielded no significant differences in growth rates and therefore no evidence for local adaptation. However, regarding the lineages, the evidence reveals that the strain affiliated with the amplus lineage exhibits significantly lower growth rates in the tested habitats compared to each of the three lineage simplex strains.

## Discussion

### Stepping stones

The K_a_/K_s_ calculations and transplantation experiments did not show signs of local adaptation. All tested strains were able to grow in water from different sites. Numerous habitats within the Alps that are ecologically similar to the tested ones could potentially serve as stepping stones for gene flow among local *P. asymbioticus* populations across distant sites. Moreover, even if dispersed bacteria go extinct in foreign habitats, their genes may still be introduced into the resident populations by recombination.

### Sexual species and mismatch repair

The estimated r/m of 7.4 for the investigated *P. asymbioticus* core genome clearly exceeds estimated thresholds for the transition from clonal to sexual species [17, 23], which is passed at r/m ratios between 0.25 and 2 according to [17]. The respective strains were isolated from habitats within a maximum distance from each other of 76 km. Gene flow concerning the core genome of *P. asymbioticus* is obviously sufficient across these habitats to provide genomic coherence of the species. Dispersal limitation may be effectively reduced by interjacent habitats that potentially serve as stepping stones in the dispersal of these bacteria. In addition, the high abundance of *P. asymbioticus* in certain habitats [48, 54] may increase the chance for an effective dispersal. Furthermore it is conceivable that the success rate of single recombination events is enhanced within this species due to the lack of the *mutS*/*mutL* mismatch repair system [74–77]. Interestingly, according to genome data this mismatch repair system seems to be intact in other species of the *Polynucloebacter* genus not affiliated with the PnecC cluster and in the closest related genera, i.e. *Cupriavidus* and *Ralstonia*, as well. An extraordinarily high diversification has been reported for the PnecC cluster, coinciding with a disproportionally high overall genome diversity in relation to 16S rRNA gene diversity [59]. It can be speculated that radiation in the PnecC cluster has been promoted in consequence of loss of the *mutS*/*mutL* system. Possibly, HR acts as the cohesive force also within other species of the PnecC cluster and consequently is responsible for the clear genomic demarcation between *Polynucleobacter* species within PnecC. It should be noted that flexible genes can blur species boundaries, as genomic islands can be transferred between different species [64]. Hence, the biological species concept might be useful for PnecC bacteria but in a less strict manner than for sexually reproducing organisms.

### HR and the evolution of prokaryotes in inland waters

Studies of HR in prokaryotes isolated from inland waters are rare. Examples are found for freshwater bacteria of the SAR11 clade, for which notably low recombination rates have been reported in contrast to the marine sister clade [46]. The freshwater SAR11 population may be affected by a putative bottleneck after the transition from marine to freshwater systems; that is, the low effective population size may be the reason for low rates of detectable recombination [46]. Similar reasons may underlie the observed clonality of many pathogens, which could be a result of their physically constrained population structure and population bottlenecks after shifts to new hosts rather than a mechanistic handicap of recombination (compare [87] and [39]).

For archaea affiliated with the genus *Sulfolobus* it has been shown that geographic gene flow barriers shape these populations. While pronounced HR has been evidenced in syntopic *Sulfolobus islandicus* subpopulations [40, 88], recombination across distant sites is effectively prevented [50, 51]. For these thermoacidophilic archaea, regions providing potential habitats are generally far apart, and hence, stepping stones for dispersal may not be available [50, 51].

Pronounced HR among geographically separated *P. asymbioticus* subpopulations as suggested in this study contrasts the specific cases of freshwater SAR11 and *Sulfolobus islandicus*. Further investigations concerning recombination in other taxa would be necessary to clarify whether this corresponds to another extreme or rather to the prevalent situation in prokaryotes of inland waters.

### Differences between MLST and genome-wide data

There is evidence that HR rates can vary greatly between different genome regions [41, 82, 84, 89], which suggests that rates determined by use of MLST data may often not represent genome-wide rates. Furthermore, as the detection of HR is usually limited to events that introduced a relatively high number of polymorphisms rather than events between very similar sequences, rates may often be underestimated (e.g. compare [90] and [91]). As shown in Fig. 4, the inferred HR frequency in *P. asymbioticus* varies considerably along the genome. In some regions positive selection pressure may tend to introduce variation. This is assumed for the genomic island related to cell surface composition (CSC in [64] and Fig. 4), where phage predation may impose selection pressure towards variation [64, 92]. Other genome regions are rather conserved, and in these regions purifying selection may act against HR. This may be particularly the case for genes which are required for basic cellular functions, which are typically sequenced for MLST [36]. Therefore, MLST data may generally be prone to underestimate genome-wide recombination rates, which is confirmed by this study. The lower HR rates are the probable cause of the higher F_ST_ values for MLST data in comparison to genome-wide data of this study. As total panmixis of the *P. asymbioticus* population across sites is precluded by dispersal limitations, genetic drift can cause differentiation among the geographically separated subpopulations. This differentiation is more likely in genomic regions which are less impacted by recombination or the MLST data, respectively. The signals of subpopulation differentiation may be transient however, and would possibly fade if strains isolated over a longer time period were analyzed.

### Flux of HR

The ClonalOrigin analysis indicated an increased flux of HR from Loi strains towards Tro strains but not vice versa. Loi is located at the foothills of the Austrian Alps, at the periphery of the *P. asymbioticus* range ([48, 57] and unpublished data), and the diversity found at this site was comparably low and stable (see [48, 62] and Fig. 2). It can be speculated that the resident *P. asymbioticus* population is less impacted by gene flow due to its peripheral location. In contrast, Tro is located more centrally, which could be the reason for a larger effective population size, higher diversity and a greater impact of recombination at this site.

Rau is the only site where isolates affiliated with the amplus lineage have been obtained. The lineage has never been detected at Loi and Tro, but it has been detected at a few other sites in the Austrian Alps; always, however, in small numbers compared to the simplex lineage (unpublished data). It is conceivable that the sampled habitats in the Alps only represent an overlap of two distinct taxon ranges of the two lineages. Thus, the amplus lineage may be more abundant in regions that have not yet been sampled, which could be different areas of the Alps or even a more distant mountain range. Recombinational export from the branch leading to the amplus genomes was found less frequently than expected. This may be explained by the low abundance of the amplus lineage compared to the simplex lineage in the sampled area. Moreover, the reduced import from the ancestral branch of the simplex genomes towards the terminal branches of the two amplus genomes could indicate a HR barrier between the two lineages. Such a barrier is also suggested by the distant phylogenetic position of the two amplus genomes from all simplex genomes in all trees in Fig. 3, and the higher r/m ratio inferred from MLST data when amplus strains are excluded from the analysis. A possible scenario could be that the two lineages inhabited different refugia during the last glacial period (ca. 110,000–12,000 years ago). In such a scenario, the lineages could now be in a process of convergence after sympatry may have been restored. Recombination has been suspected of causing convergence in other taxa, after recombination barriers between formerly separated lineages had disappeared [93, 94]. Alternatively, amplus and simplex strains may have diverged too much for subsequent cohesion by HR, and the divergence between the lineages may thus be ongoing. This would give rise to speciation between the two lineages. Incipient speciation is also conceivable in a scenario where the HR barrier between the two lineages was not a result of geographic but of ecological separation. Moreover, geographic and ecological separation may go hand in hand. Higher K_a_/K_s_ ratios in comparisons between the lineages relative to comparisons within simplex point to a selective divergence, which may be expected in scenarios of both geographical and ecological separation.

## Conclusions

Different *Polynucleobacter* species affiliated with the globally abundant PnecC cluster can be clearly delineated by whole genome comparisons. High HR rates in *P. asymbioticus* demonstrate that recombination can play an important role in the evolution of bacterial species inhabiting geographically separated freshwater habitats, at least on a regional scale. It is suggested that HR acts as the cohesive force that effectively counteracts genetic divergence between *P. asymbioticus* subpopulations. A species model resembling the biological species concept may therefore be applicable to these bacteria. Divergence between two lineages of *P. asymbioticus* coincides with decreased HR between these lineages. To learn more about potential recombination barriers and possible speciation between the two lineages, further isolation of respective strains from different regions would be necessary.

## Methods

### *P. asymbioticus* strains

The 37 *P. asymbioticus* strains were isolated over eleven years from nine habitats at three different sites (Loi, Rau and Tro) in the Austrian Alps, as has been described elsewhere [64]. Data on the strains is given in Additional file 1 and data on the sites of origin in Additional file 8. All 37 strains have been characterized earlier by multilocus sequence typing (MLST) of eleven loci [48]. The genomes of nine strains, three from each aforementioned site, have been sequenced, assembled and annotated in a previous study [64]. Further characterization of the strains and a detailed analysis on their flexible genome can be found in [64].

### PnecC genomes analyses

Twenty strains affiliated with the PnecC cluster have been whole genome sequenced in previous studies [60, 64–70]. Data regarding these strains is given in Additional file 1. ANI values for all pairwise comparisons of the 20 PnecC genomes were calculated using the IMG/ER comparative analysis system [78] (Fig. 1 and Additional file 2). The IMG/ER system was also used to identify homologous genes among the genomes (Additional file 9).

### Screening for *mutL* and *mutS*

Genomes affiliated with PnecC and its closest related taxa were screened for the presence/absence of the genes *mutL* and *mutS* using the IMG/ER analysis system [78]. The dataset included 22 *Polynucleobacter* genomes, i.e. 20 PnecC, one PnecA and one PnecD genome. Furthermore, 29 *Cupriavidus* and 65 *Ralstonia* genomes were obtained from the IMG/ER public database after excluding single-cell genomes.

### Phylogenetics

For the unrooted tree in Fig. 2 the nucleotide sequences of the eleven MLST loci were concatenated (7259 bp) and aligned in MEGA7 [95] by Muscle [96]. A DNA substitution model analysis was conducted in MEGA7, and the model with the lowest BIC score was selected for Maximum Likelihood tree calculation, i.e. the Tamura 3-parameter model [97] and a discrete Gamma distribution (5 categories), to model evolutionary rate differences among sites. Bootstrap values were calculated from 100 bootstrap replications.

The trees in Fig. 3 are based on a multiple whole genome alignment of the nine *P. asymbioticus* genomes performed by progressiveMauve [98, 99]. The alignment was refined with Gblocks v0.91b [100] using default parameters to omit poorly aligned positions. The resulting alignment of 1.81 Mbp was split into nine consecutive segments of equal length. For each segment an unrooted maximum-likelihood based RAxML [101] tree was inferred using the Cipres Science Gateway V.3.3 [102]. The GTRCAT model with 25 distinct rate categories was used.

Incongruences among the trees in Fig. 3 were determined using the Shimodaira-Hasegawa test [72]. The test was executed for each of the nine segments. In each test the log-likelihood for each of the nine tree topologies was calculated given the sequence alignment data of the segment. The tests were conducted in R [103] using the package phangorn 1.99–13 [104].

### Population differentiation

Similarly as in [64], the strains were grouped according to geographic origin Loi, Rau and Tro and according to lineage (simplex and amplus), respectively. F_ST_ values were calculated based on pairwise differences (pi) in Arlequin 3.5.2.2 [105]. Significances of F_ST_ values were determined through comparisons to a permutation test, in which 10,000 permutations and *p* < 0.1 were considered significant.

### Inference of HR

HR regarding MLST data was inferred using ClonalFrame [71] similarly as described in [36]. ClonalFrame estimates the clonal genealogy of a sample of strains based on a coalescent model [106]. By attempting to reconstruct the mutation and recombination events that took place on the branches of this genealogy, several evolutionary parameters are estimated, including the r/m ratio. The analysis was performed for two datasets, one including both simplex and amplus strains and the other including only simplex strains. Only one representative of each sequence type was included to avoid underestimating r/m ratios, as recombination between identical sequences cannot be detected. Thereby, also possible effects from transient blooming of certain genotypes are precluded. For both datasets, two runs of the ClonalFrame Monte Carlo Markov chain (MCMC) were performed, each consisting of 2,000,000 iterations. The first half of the chains was discarded and the second half was sampled every hundred iterations. Convergence of the MCMC for the two runs of each dataset was confirmed by the Gelman-Rubin convergence test [107]. For each dataset only the results of the run with the higher average log-likelihood are given in Table 1. The μ/ρ ratio as it is used in [23] was calculated as π (r/m)^−1^. The nucleotide diversity π for each dataset was calculated using DnaSP 5.10.1 [108].

For inference of HR in the core genome of *P. asymbioticus*, the process as described in [84] was followed. At first, the nine genomes were aligned by progressiveMauve [98, 99]. The stripSubsetLCBs script was used to leave only alignment blocks longer than 500 bp. A total of 111 such blocks was obtained, ranging in size from 607 bp to 116,266 bp with a concatenated length of 1,814,913 bp (Additional file 4). These 1.8 Mbp represent the core genome of the nine *P. asymbioticus* strains. Three runs of the ClonalFrame MCMC were performed on this core genome alignment, each consisting of 1,010,000 iterations. The first 10,000 iterations were discarded and the subsequent 1,000,000 iterations were sampled every ten iterations. Again, convergence of the runs was confirmed by the Gelman-Rubin convergence test [107]. By comparing the output trees it was verified that the three runs produced a consistent genealogy. Based on this genealogy, further properties of HR were inferred using ClonalOrigin [84], which performs approximate inference under the coalescent model with gene conversion [109]. ClonalOrigin infers the origin and destination of recombination events on the genealogy, as well as the three parameters δ (average length of recombination events), θ_s_ (scaled mutation rate) and ρ_s_ (scaled recombination rate). The ClonalOrigin MCMC was run independently on each of the 111 blocks of the core genome alignment specified above. The values of δ, θ_s_ and ρ_s_ for each block were calculated by the computeMedians.pl script [84] (Additional files 4 and 10). The same script was used to calculate the weighted median values of these parameters across all blocks, at which the following values were obtained: δ = 2340 bp, θ_s_ = 0.0109, and ρ_s_ = 0.0056. ClonalOrigin was then rerun for each block with the three parameters set equal to these estimates. Both ClonalOrigin runs were performed with 2,000,000 iterations of the MCMC chain, the first half of which discarded and the second half sampled every 10,000 iterations. The data used in Fig. 4 and Fig. 5 was extracted from the output of the second run using the ClonalOrigin GUI.

### K_a_/K_s_ calculations

The core genes of the nine *P. asymbioticus* genomes were identified as follows. A blast was performed using the IMG/ER comparative analysis system [78], including all protein coding genes of one genome as reference (QLW-P1DMWA-1 was chosen arbitrarily) and each other genome as query. Best blast hits for each genome were retained, applying a threshold >70% amino acid sequence identity. For query genes present multiple times in the obtained dataset, only the hit with the highest bit score was kept. The resulting dataset comprised 1820 reference genes with hits in all query genomes, which were defined as core genes (Additional file 9). Stop codons were removed from the core genes of all nine strains. The gene sequences of each strain were concatenated such that homologous genes were in the same order for all strains. From these concatenated nucleotide sequences the respective amino acid sequences were generated using EMBOSS Transeq [110]. The amino acid sequences of all pairwise combinations of strains were aligned with MAFFT version 7 [111]. The pal2nal.pl script [112] was used to generate the codon alignments from the amino acid alignments and the respective nucleotide sequences. Axt files were generated using the parseFastaIntoAXT.pl script [113] and the K_a_/K_s_ ratios were calculated from these files for pairwise comparisons of strains using the K_a_/K_s_ calculator 2.0 [114] and the γ-MYN method [115]. The strains QLW-P1DMWA-1 and P1–4-10KL differ by only 15 SNPs within the core genes and were therefore excluded from the analysis described in the following. The pairwise K_a_/K_s_ ratios within the simplex strains as well as the ratios between simplex and amplus strains were grouped together and tested for normal distribution by the Shapiro-Wilk test. The test failed for both groups on a significance level *p* = 0.05. The Mann-Whitney rank sum test was performed to test for significant differences of K_a_/K_s_ ratios between the two groups.

### Transplantation experiments

Reciprocal transplantation of strains was conducted to study growth performance of strains in sterile water of their home habitats (“home”) and in sterile water of the home habitats of the other strains included (“away”). The experiments were performed with four pure culture strains, two of which were isolated from Rechteckteich in Rau (MWH-Recht1 and MWH-RechtKolB), one from Trog-7 in Tro (MWH-Tro7–1-4) and one from Trog-8, also in Tro (MWH-Tro8–2-9). Two experiments were conducted, the first in August 2014 and the second in September/October 2014. Water from Rechteckteich, Trog-7 and Trog-8 [64] was collected on 19th August and 25th September 2014 and sterilized by 0.1 μm filtration. The experiment was conducted in 50 ml cultures in agitated Erlenmeyer flasks at 15 °C in the dark. The tested strains were pre-cultured for eight days in diluted (1 g l^−1^) NSY medium [63], with an initial pH of 5.8. Subsequently, they were pre-cultured in the water in which the experiment was conducted for four days. The strains were inoculated into fresh medium/water several times during pre-cultivation. The experiment was initiated by transferring the pre-cultivated strains during the exponential growth phase into fresh water to yield a starting concentration of about 10^5^ cells/ml. All four strains were tested in parallel in the three waters (triplicates each). Growth was monitored for 139 and 48 h, in the first and second experiment, respectively. Bacterial numbers were determined by flow cytometry. To ensure that growth rates were determined within the exponential growth phase, only the first 15 h of the experiment were consulted for growth rate calculations. For this purpose, three samples taken at approximately seven-hour intervals and four samples taken at approximately four-hour intervals were analyzed in the first and second experiment, respectively. The growth rate for each culture was estimated as the slope of the logarithm of cell concentration versus time, determined by linear regression.

All doubling times (Log(2) / growth rate) regarding a certain strain were grouped together and tested for normal distribution by the Shapiro-Wilk test. Furthermore the doubling times for “home” as well as those for “away” were grouped together and tested for normality. The test failed for the strains as well as for “home” and “away” on a significance level *p* = 0.05. The Mann-Whitney rank sum test was performed to test for significant differences of doubling times between the strains or between home and away, respectively.

## Additional files

Additional file 1: List of all PnecC strains used in this study. (XLSX 17 kb)

Additional file 2: ANI values for all PnecC genome pairs. (XLSX 26 kb)

Additional file 3: 16S rRNA tree of *Polynucleobacter*. (PDF 61 kb)

Additional file 4: Core alignment blocks. The 111 blocks obtained from the alignment of the nine *P. asymbioticus* genomes are listed. Respective parameters obtained from the two ClonalOrigin runs are given. The last column gives the segment number of the split alignment (Fig. 3 and Table 2) in which the respective blocks are contained. (XLSX 24 kb)

Additional file 5: Inferred numbers of events between branches of the genealogy. The number of events as inferred from the second ClonalOrigin run and used in Fig. 5 are given. Observed numbers, expected numbers and their ratios are given in the first, second and third sheet, respectively. (XLSX 20 kb)

Additional file 6: Ka/Ks values for the alignment of the 1820 core genes for all *P. asymbioticus* genome pairs. (XLSX 16 kb)

Additional file 7: Growth curves of the two transplantation experiments performed with four *P. asymbioticus* strains from three different habitats. (PDF 62 kb)

Additional file 8: Characterization of the nine habitats from which the 37 *P. asymbioticus* strains have been isolated. (XLSX 11 kb)

Additional file 9: Homologous genes among the 20 PnecC genomes. The genome with the highest number of genes (MWH-RechtKol4) was used as a reference. For each of the 2362 protein coding genes of MWH-RechtKol4, the presence/absence of a homologous gene in each of the 19 other genomes was determined by applying an amino acid identity threshold of 70%. The number of reference genes for which a homolog is found in the respective query genome is given in the second row. It should be noted that these numbers can be slightly higher than the actual number of homologous genes in the query, as certain query genes may be counted twice when compared to paralogs of the reference. When considering each gene only once, 1820 *P. asymbioticus* core genes have been identified, which are highlighted in the first column. (XLSX 245 kb)

Additional file 10: Parameters inferred from the first ClonalOrigin run. The parameters δ, θ_s_ and ρ_s_ are plotted along the genome. The computed median values, which have been used in the second ClonalOrigin run, are indicated by red, dashed lines. (PDF 28 kb)

## Abbreviations

ANI: Average nucleotide identity
bp: Base pairs
F_ST_: Fixation index
HR: Homologous recombination
K_a_/K_s_: Ratio of the number of non-synonymous substitutions per non-synonymous site (K_a_) to the number of synonymous substitutions per synonymous site (K_s_)
Loi: Loibersbacher Höhe site
MCMC: Monte Carlo Markov chain
MLST: Multilocus sequence typing
*P. asymbioticus*: *Polynucleobacter asymbioticus*
PnecC: *Polynucleobacter* subcluster C
r/m: Ratio of substitutions introduced by recombination relative to mutation
Rau: Rauriser Urwald site
Tro: Trög site
