# Supplementary material for: Genomic divergence and cohesion in a species of pelagic freshwater bacteria
Source: BMC Genomics. 2017 Oct 16;18:794. doi: 10.1186/s12864-017-4199-z (PMC5644125; doi:10.1186/s12864-017-4199-z)
Supplement: Supplementary file 3 — 16S rRNA tree of Polynucleobacter. (PDF 61 kb) [file 12864_2017_4199_MOESM3_ESM.pdf]

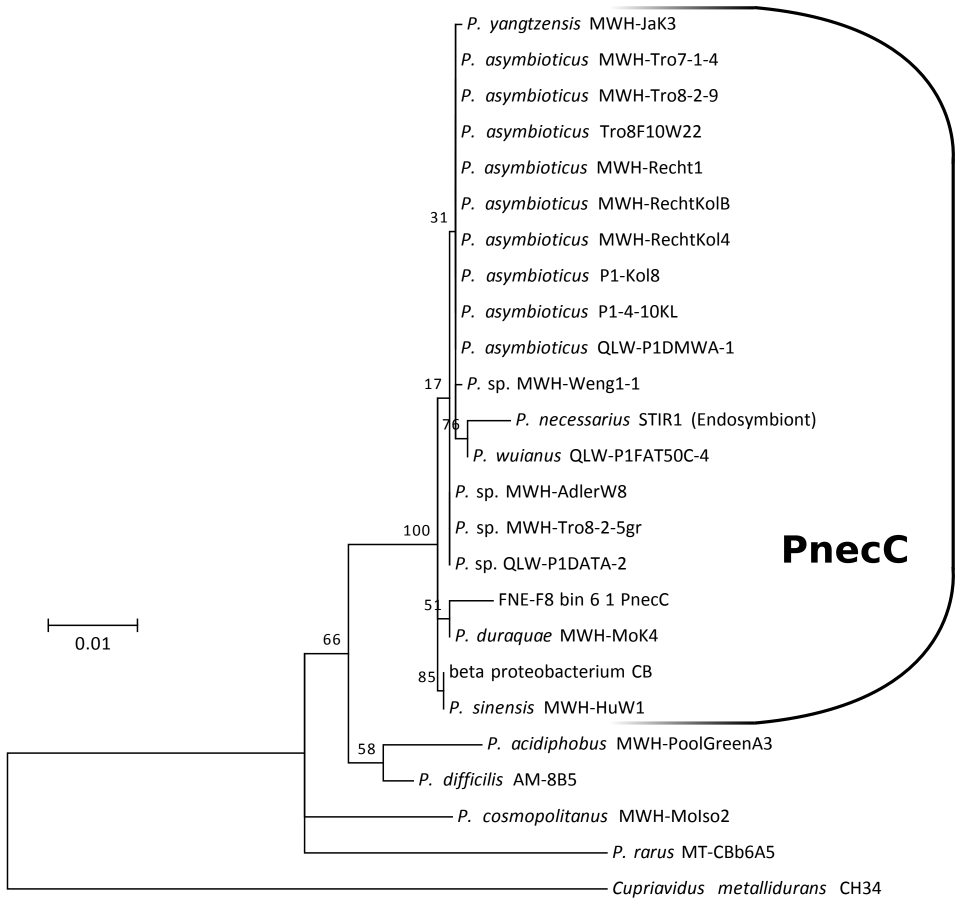

Additional file 2. Unrooted Maximum Likelihood tree based on partial 16S rRNA gene sequences. The tree was inferred by using the Kimura 2-parameter model [1]. The percentage of trees in which the associated taxa clustered together is shown above the branches. A discrete Gamma distribution was used to model evolutionary rate differences among sites (5 categories (+G = 0.0536)). The rate variation model allowed for some sites to be evolutionarily invariable ([+I], 35.6880% sites). The tree is drawn to scale, with branch lengths measured in the number of substitutions per site. There were a total of 1494 positions in the dataset. Evolutionary analyses were conducted in MEGA7 [2].

1. Kimura M. (1980). A simple method for estimating evolutionary rate of base substitutions through comparative studies of nucleotide sequences. Journal of Molecular Evolution 16:111-120.
2. Kumar S., Stecher G., and Tamura K. (2016). MEGA7: Molecular Evolutionary Genetics Analysis version 7.0 for bigger datasets. Molecular Biology and Evolution 33(7):18701874.
