# Supplementary figures and images for: Genomic divergence and cohesion in a species of pelagic freshwater bacteria
Source: BMC Genomics. 2017 Oct 16;18:794. doi: 10.1186/s12864-017-4199-z (PMC5644125; doi:10.1186/s12864-017-4199-z)

**August 2014**

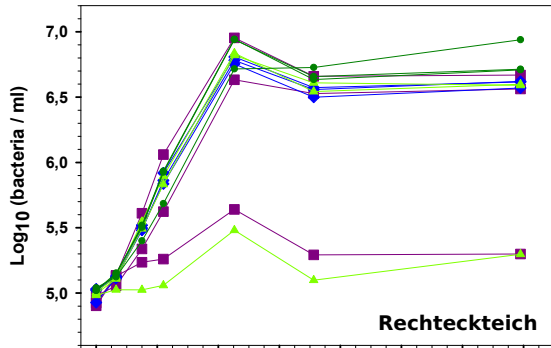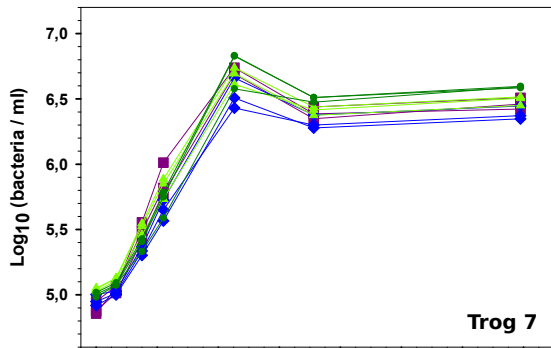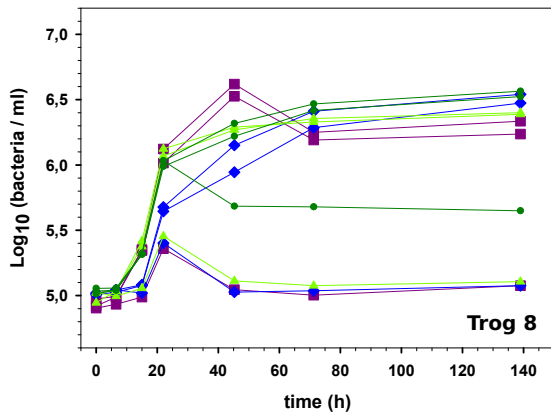

**September/October 2014**

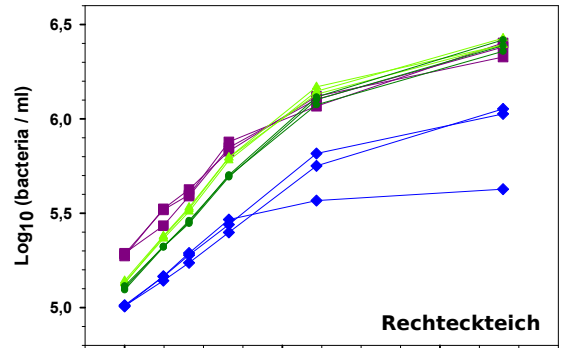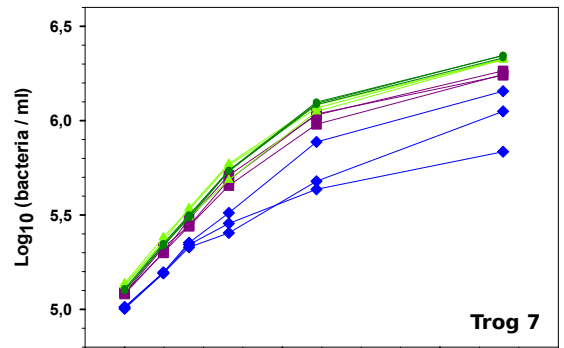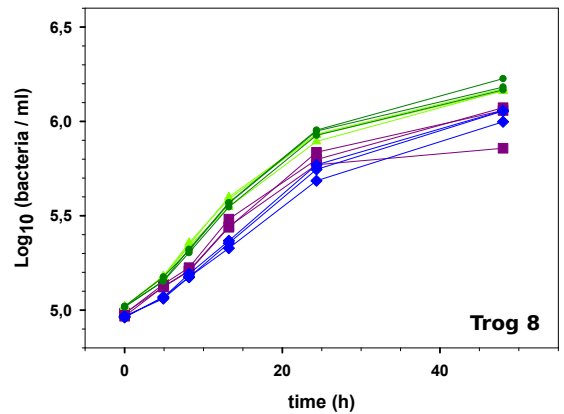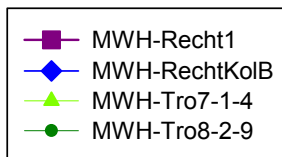

**Additional file 5.** Transplantation experiments, growth curves

Supplement: Supplementary file 7 — Growth curves of the two transplantation experiments performed with four P. asymbioticus strains from three different habitats. (PDF 62 kb) [file 12864_2017_4199_MOESM7_ESM.pdf]

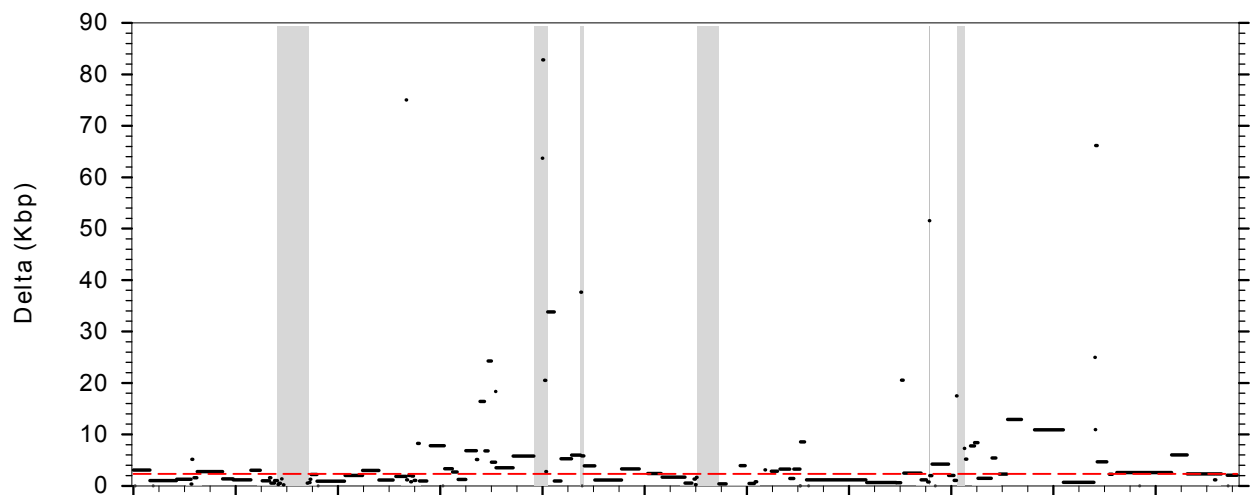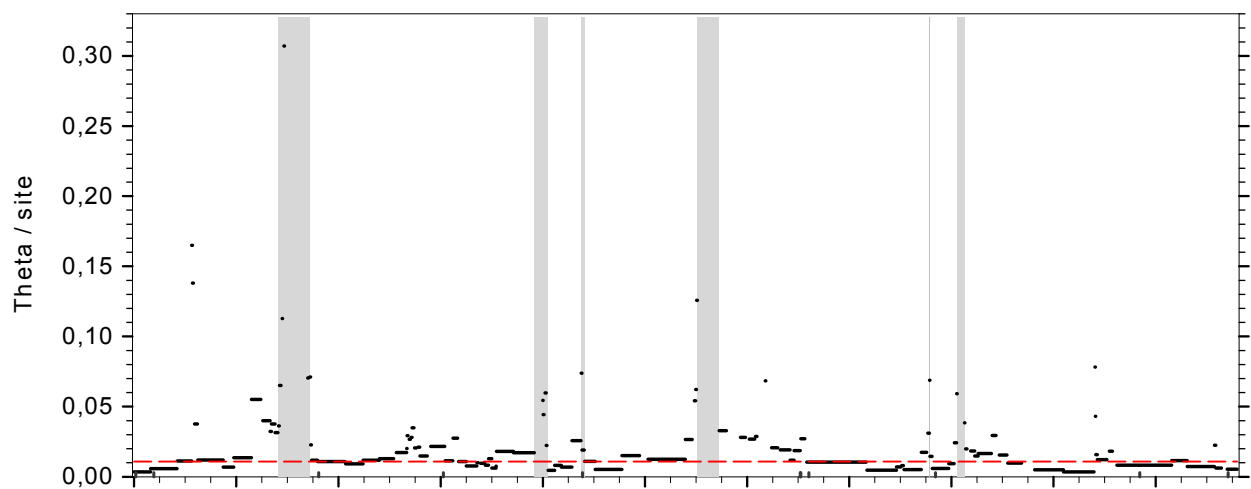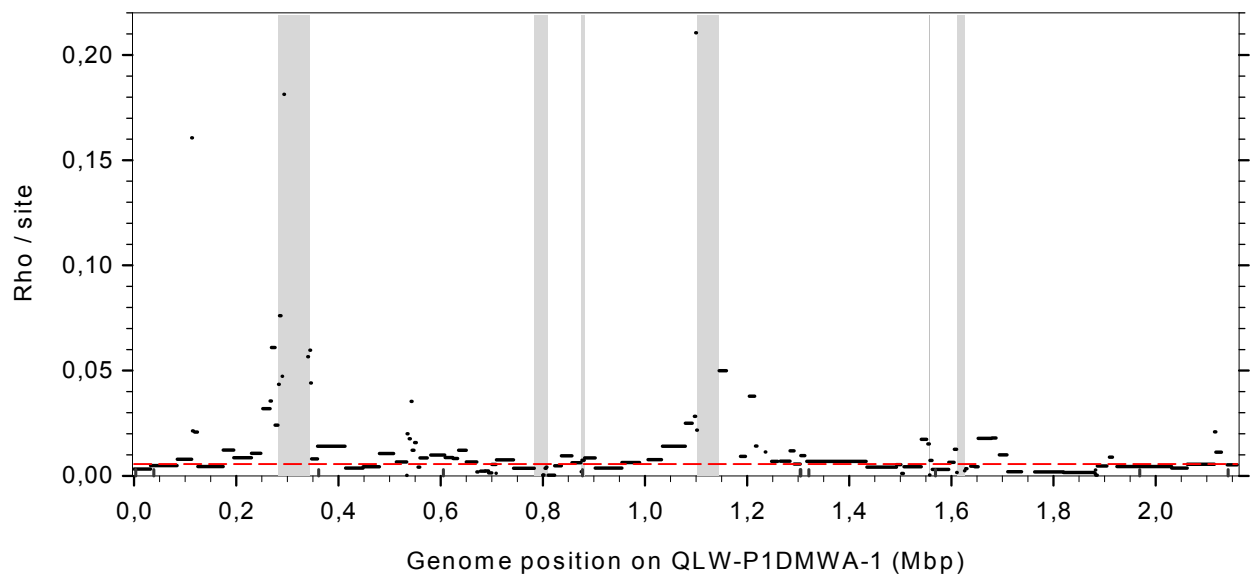

Supplement: Supplementary file 10 — Parameters inferred from the first ClonalOrigin run. The parameters δ, θs and ρs are plotted along the genome. The computed median values, which have been used in the second ClonalOrigin run, are indicated by red, dashed lines. (PDF 28 kb) [file 12864_2017_4199_MOESM10_ESM.pdf]
